# Explicit and Implicit Components of the Emotional Processing in Non-organic Vision Loss: Behavioral Evidence About the Role of Fear in Functional Blindness

**DOI:** 10.3389/fpsyg.2018.00494

**Published:** 2018-04-10

**Authors:** Federica Scarpina, Lisa Melzi, Gianluca Castelnuovo, Alessandro Mauro, Stefania B. Marzoli, Enrico Molinari

**Affiliations:** ^1^Psychology Research Laboratory, Ospedale San Giuseppe, IRCCS Istituto Auxologico Italiano, Milan, Italy; ^2^Neuro-Ophthalmology Service and Electrophysiology Laboratory, Department of Ophthalmology, Scientific Institute Capitanio Hospital, Istituto Auxologico Italiano, Milan, Italy; ^3^Department of Psychology, Università Cattolica del Sacro Cuore, Milan, Italy; ^4^“Rita Levi Montalcini” Department of Neuroscience, University of Turin, Turin, Italy; ^5^Division of Neurology and Neuro-Rehabilitation, Ospedale San Giuseppe, IRCCS Istituto Auxologico Italiano, Milan, Italy

**Keywords:** non-organic visual loss, facial emotion recognition, redundant target effect, fear, alexithymia, visual perception

## Abstract

Non-organic vision loss (NOVL), a functional partial or global vision loss, might be considered a manifestation of conversion disorder. The few previous studies focused on investigating the relationship between cerebral activity and subjective symptoms in NOVL; however, the emotional processing is still neglected. In the present case-controls study, we investigated the capability of two individuals diagnosed with NOVL to recognize implicitly the emotions of fear and anger; this was assessed through a facial emotion recognition task based on the redundant target effect. In addition, the level of alexithymia was measured by asking them to judge explicitly their ability to identify and describe emotions. Both individuals showed selective difficulties in recognizing the emotion of fear when their performance was contrasted with a matched control sample; they also mislabeled other emotional stimuli, judging them as fearful, when they were not. However, they did not report alexithymia when measured using a standard questionnaire. This preliminary investigation reports a mismatch between the implicit (i.e., the behavior in the experimental paradigm) and the explicit (i.e., the subjective evaluation of one’s own emotional capability) components of the emotional processing in NOVL. Moreover, fear seems to represent a critical emotion in this condition, as has been reported in other psychiatric disorders. However, possible difficulties in the emotional processing of fear would emerge only when they are inferred from an implicit behavior, instead of a subjective evaluation of one’s own emotional processing capability.

## Introduction

Non-organic vision loss (NOVL) is a functional partial or global vision loss in which an organic disease or a pathology in the visual system does not explain a subjective visual disturbance (Beatty, 1999; Stone et al., 2005; Bruce and Newman, 2010). Based on this description, NOVL might be considered a manifestation of a conversion disorder (Stone et al., 2005; World Health Organization, 2007; Bruce and Newman, 2010). Visual complaints without a physical basis are frequently presented to the clinicians (Beatty, 1999) and the clinical diagnosis is generally formulated according to the results from an extensive neuro-ophthalmic evaluation. To our knowledge, few studies investigating NOVL have been reported in the literature: they focused on the relationship between cerebral activity and subjective symptoms (Werring et al., 2004; Schoenfeld et al., 2011) in affected individuals, while an extensive investigation of the psychological and emotional components has been lacking. In a single case study by Becker et al. (2013), a relationship between the activity of the cerebral occipital lobe and cerebral areas implicated in emotional regulation and moral reasoning was preliminarily sketched out; however, the authors did not investigate extensively the affected individual’s behavior upon recognizing emotional stimuli.

In this work, we provide for the first time in the literature, to current knowledge, an investigation of the implicit and explicit components of the emotional processing in two individuals affected by NOVL. To study their capability to implicitly recognize emotions expressed by others, we adopted a facial emotion recognition task based on the “redundant target effect” (Miniussi et al., 1998), according to which people respond faster when two identical targets are presented simultaneously rather than when presented alone. Moreover, the competitive presence of a non- identical stimulus (i.e., the distractor) affects the efficient recognition of the target, with an increase of velocity and a reduction of the level of accuracy. Since this effect occurs in early visual processing rather than in later (decisional or premotor) stages (Miniussi et al., 1998), this method appears suitable for indirectly investigating residual affective recognition in (alleged) blind sight: it preserves participants from making any explicit counterintuitive guesses about unseen events in the blind area (de Gelder et al., 2001). Thus, the individuals’ emotional capability is inferred from their behavior in the task. Moreover, following the previous results reported in the literature about aberrant cerebral activity in NOVL (Werring et al., 2004; Schoenfeld et al., 2011), we also studied the individuals’ capability to recognize neutral visual stimuli (i.e., geometrical shapes).

On the other hand, the explicit component of the emotional processing was investigated. We focused not only on depressive, anxiety and different psychopathological symptoms and quality of life, but also on the level of alexithymia, using the Toronto Alexithymia Scale 20 (TAS-20) (Bagby et al., 1994; Todarello and Pace, 2010). Focusing on the relationship between somatization and alexithymia, the latter is generally associated with reports of medically unexplained symptoms (Bach and Bach, 1996; Waller and Scheidt, 2006; Mattila et al., 2008; Demartini et al., 2014) and it is frequently observed in various psychiatric disorders especially in the somatoform ones (Demartini et al., 2014; Gulpek et al., 2014). Alexithymia means difficulty in identifying and describing one’s own emotions, the tendency to minimize emotional experience and to focus attention externally (Sifneos, 1973). Since this concept refers to the cognitive processing of emotions, instead of subjective intrapsychic conflicts that possibly generate bodily symptoms in psychosomatic disease (Taylor et al., 1991), it is suitable when participants’ self-ability to recognize their own emotions is studied independently of the subjective causes of the emotional difficulties, as in the present study.

Embracing the description of NOVL as a somatoform disorder (Stone et al., 2005; World Health Organization, 2007; Bruce and Newman, 2010), we would expect to find that affected individuals have difficulties in emotional processing, with possible dissociation between the explicit and implicit components.

## Materials and Methods

The present study was approved by the Ethical Committee of the I.R.C.C.S Istituto Auxologico Italiano, Milan, Italy and performed in accordance with the ethical standards of the Declaration of Helsinki. All participants provided a written informed consent.

### Neuro-Ophthalmological Evaluation

NOVL is a neuro-ophthalmological diagnosis based on clinical and electrophysiological tests, and neuroimaging exams, which demonstrate the organic integrity of the afferent visual system (Thompson, 1985). The typical diagnostic approach included an in-depth examination to carefully exclude neurological diseases (Stone et al., 2005; Bruce and Newman, 2010), assuming the lack of pathological results is either due to an inexistent pathology or because the adopted measures were not sensitive enough to detect pathology (Schoenfeld et al., 2011). Thus, the disease reported by two participants is judged as functional until proven otherwise. SM and LM (co-authors of the present manuscript) had the specific role of conducting the extensive evaluation of the two cases. When assessing the patients, the indications provided by Bruce and Newman (2010) were followed, collecting detailed patients’ medical history: this allowed physicians to appropriately localize potential organic lesions and to guide the neuro-ophthalmological examination. First of all, a complete neuro-ophthalmological examination was performed [best corrected visual acuity; color vision (Ishihara plates); external examination of eyes, orbits and lids, ocular motility, slit lamp examination for intraocular pressure; pupillary reactions; dilated fundus examination]. After, the diagnostic approach required differentiating monocular from binocular visual loss and central visual loss from peripheral visual loss, through the Humphrey test of visual fields with the Swedish Interactive Threshold Algorithm 30-2 (Carl Zeiss Meditec, Dublin, CA, United States). Moreover, the SD-OCT - Spectral Domain Optical Coherence Tomography (SD-OCT) imaging (RTVue-100 Version 5.1, Optovue Inc. Fremont, CA, United States) was used to exclude any structural damage. The functional integrity of the afferent visual system was assessed through electrophysiological exams (visual evoked potentials, pattern electroretinogram, full field electroretinogram and multifocal electroretinogram) and magnetic resonance imaging of the optic nerve. Also, magnetic resonance imaging of the whole brain allowed exclusion of any cerebral lesions. Finally, the absence of any anamnestic reports of neurological disease, tumor or TBI was verified during the collection of the medical history.

**FIGURE 1:**
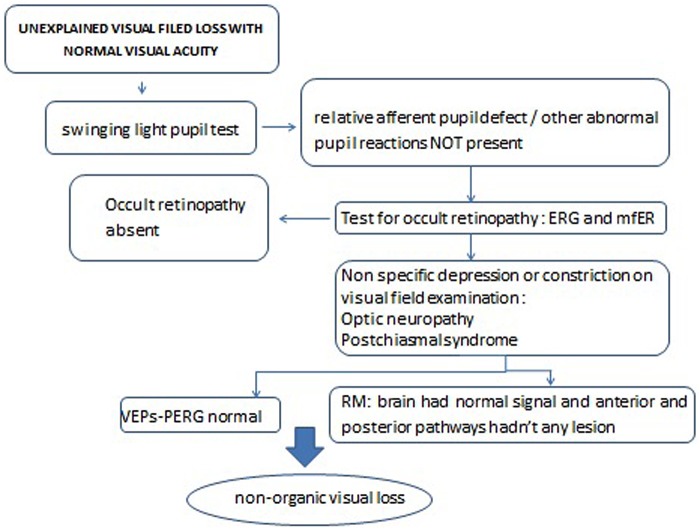
Decision tree in the case of “unexplained visual field loss with normal visual acuity.”

SM and LM followed the diagnostic decision tree for unexplained visual field loss with normal visual acuity (**Figure 1**), in order to exclude other possible medical causes for a visual field loss with normal visual acuity, reported in **Table 1**.

**Table 1:** About the diagnostic process, other causes and test to exclude them are reported.

Cause of visual field loss with normal visual acuity	Clinical evidences	Instrumental evidences	Conclusion
Retinopathy	Manifest	- No ophthalmoscopy lesion	Manifest retinopathy absent
		- Macula normal on OCT.	
	Occult	- Normal ERG	Occult retinopathy absent
		- Normal mfERG	
	*No clinical evidence*	*No instrumental evidence*	
Optic neuropathy	- Normal optic nerve ophthalmoscopic appearance	- Normal Neuroimaging	Optic neuropathy absent
		- Normal VEP	
	- Normal peripapillary retinal nerve fiber layer thickness values by three-dimensional OCT	- Normal PERG	
	*No clinical evidence*	*No instrumental evidence*	
Retrochiasmal lesion	-Normal optic nerve at the ophthalmoscopic appearance	- Normal Neuroimaging	Retrochiasmal lesion absent
		- Normal VEP	
	- Normal peripapillary nerve fiber layer macular complex ganglion cell thickness on OCT	- Normal PERG	
	*No clinical evidence*	*No instrumental evidence*	


More details about the clinical assessment to exclude the presence of a clinically recognizable disease are reported in the following sections. The organic integrity of the afferent visual system was then verified through the objective measurements (clinical and electrophysiological tests, and neuroimaging), leading to the neuro-ophthalmological diagnosis of NOVL.

### Psychiatric Evaluation

GC and EM (co-authors of this work) conducted independently a psychiatric evaluation of both patients before the experimental procedure, according to the Italian version of the Structured Clinical Interview for Axis I Disorders (SCID-I) (First et al., 1996). The evaluation led to the same diagnostic conclusion for both patients, that is, a diagnosis of “Conversion disorder with sensory symptom or deficit” [F44.6], according to the nosographical approach (World Health Organization, 2007).

### Participants

Case #1 and case #2 were two female individuals admitted to the Neuro-Ophthalmology Service and Electrophysiology Laboratory, Department of Ophthalmology, Scientific Institute Capitanio Hospital, Istituto Auxologico Italiano Milan, Italy for diagnostic evaluation, since they both reported having visual difficulties. However, the two patients had normal bilateral best corrected visual acuity and mild or absolute loss of peripheral vision.

### Case #1

The first case was a 37-year-old woman with 13 years of education. She was right handed. She reported a 2-year history of subacute painless vision loss in both eyes, photophobia, and ocular discomfort. The family history was negative for visual impairment. She did not report any history of previous neurological disease, tumor or traumatic brain injury. At the psychiatric evaluation, she denied any concurrent psychological issues. The patient smoked cigarettes. Co-existence of internal-medicine diseases (acute lymphoblastic leukemia treated with bone marrow transplant, systemic arterial hypertension, Hashimoto’s thyroiditis and paraparesis: flaccid legs with plausible functional limitations) was independent of visual field loss – because of the evidence of the organic integrity of the afferent visual system. Indeed, regarding her vision functionality, she had moderate myopia in both eyes that was adequately corrected. According to the complete neuro-ophthalmological examination, best corrected visual acuity (BCVA) was 20/20, and her color vision (Ishihara plates) was moderately impaired on the red-green axis. The external examination of eyes, orbits, and lids was normal. Ocular motility was normal without strabismus or nystagmus; ductions, visual pursuit, and saccades were normal. Slit lamp examination revealed a normal anterior segment and normal intraocular pressure. Pupillary reactions were normal without afferent pupillary defect. Dilated fundus examination was normal in both eyes, except for a large choroidal paramacular nevus in the left eye. The Humphrey visual field test (HVF) with the Swedish Interactive Threshold Algorithm 30-2 (Carl Zeiss Meditec, Dublin, CA, United States) revealed absolute concentric loss of peripheral vision in both eyes with the field constricted to 10° centrally (mean deviation score of –22.10 dB for the right eye and of –26.00 dB for the left eye): this result was in line with the literature, according to which, the most common visual field complaint is that related to concentric loss of peripheral vision, like “tunnel vision” (Bruce and Newman, 2010). The spectral Domain Optical Coherence Tomography (SD-OCT) imaging (RTVue-100 Version 5.1, Optovue Inc. Fremont, CA, United States) revealed a normal peripapillary nerve fiber layer, macular ganglion cell complex thickness, and macular volume and structure in both eyes. She underwent pattern visually evoked potentials (p-VEP), pattern electroretinogram (PERG), full field electroretinogram (ffERG), and multifocal electroretinogram (mfERG), which overall showed functional integrity of the afferent visual system. Brain and optic nerve MR imaging was normal, with no evidence of lesions of the anterior and posterior visual pathways. At the follow-up, performed 6 years from the onset of symptoms and during her participation in the present experiments, symptoms and signs remained unchanged, without evidence of organic afferent visual system damage.

### Case #2

The second case was a 48-year-old woman with an 8-year education. She was right handed. She reported subacute painless vision loss in her left eye, slow movement of the eyes, and ocular discomfort for 18 months before the neuro-ophthalmological examination. The family history did not indicate any problems; she did not report any history of previous neurological disease, tumor or traumatic brain injury. She was not a smoker. During the psychiatric evaluation, she denied any concurrent psychological issues. Co-existence of internal-medicine diseases (diagnosis of rheumatoid arthritis in 2006 and gastric banding for obesity with significant weight loss in 2014) were independent of visual field loss because of the evidence of the organic integrity of the afferent visual system. Indeed, she had mild bilateral astigmatism, not properly corrected. Her BCVA (20/20) and color vision were normal in both eyes; no afferent pupillary defect was observed in the affected eye. The external examination of orbits, slit lamp evaluation of the anterior segment, and intraocular pressure were normal in both eyes. Pupillary reactions and ocular motility were normal. Dilated fundus examination did not reveal any pathological changes in either eye. HVF revealed loss of peripheral vision in the right eye and mild and absolute loss of peripheral vision in the left eye (Cloverleaf visual field: mean deviation score of –10.95 dB for right eye and of -21.53 dB for left eye); this result is in line with the most common visual field complaints related to a concentric loss of peripheral vision, like “tunnel vision” (Bruce and Newman, 2010). SD-OCT did not show any changes in optic nerve or macular parameters. P-VEP, PERG, and ffERG revealed normal retinal function and optic nerve conduction. Brain neuroimaging showed only a few non-specific areas of altered signal in the frontal subcortical white matter. MRI did not show pathological changes in the orbits or optic nerves.

### Experimental Procedure

The procedure was conducted by FS, co-author of the work.

### Control Group

Twenty-five right-handed healthy volunteers (16 women, *Age M* = 42 years; *SD* = 14; range: 23–61, *Education M* = 15; *SD* = 2; range: 8–18) participated in this study. They all reported normal or corrected-to-normal visual acuity and no history of neurological or psychiatric illness.

### Psychological Assessment

After the experimental task, the participants completed self-report questionnaires. The Beck Depression Inventory (BDI) (Beck et al., 1961; Ghisi et al., 2006) was used to measure the presence of depressive symptoms. The State-Trait Anxiety Inventory (STAI) was used to measure state- and trait- anxiety (Spielberger et al., 1983; Macor et al., 1990). The Symptom Checklist 90-R (SCL-90) (Derogatis and Savitz, 2000) was used to assess the presence of psychopathological symptoms, while the Toronto Alexithymia Scale 20 (TAS-20) (Bagby et al., 1994; Todarello and Pace, 2010) was adopted to measure the level of alexithymia. No participant reported difficulties or required assistance in reading.

### The Experimental Task

The experiment consisted of two tasks, the first was a recognition *go-no go* task of neutral visual stimuli, while the second involved a recognition of emotional visual stimuli. For both tasks, the participants were seated at a distance of ∼60 cm from a computer screen of which the vertical midline lay on the sagittal midplane of their trunk and head. They had to press the spacebar of a keyboard with their dominant hand to answer the questions according to the instructions. All participants completed the experimental test without any complaints about difficulties in their ability to look at the screen.

### Non-emotional Task

**FIGURE 2:**
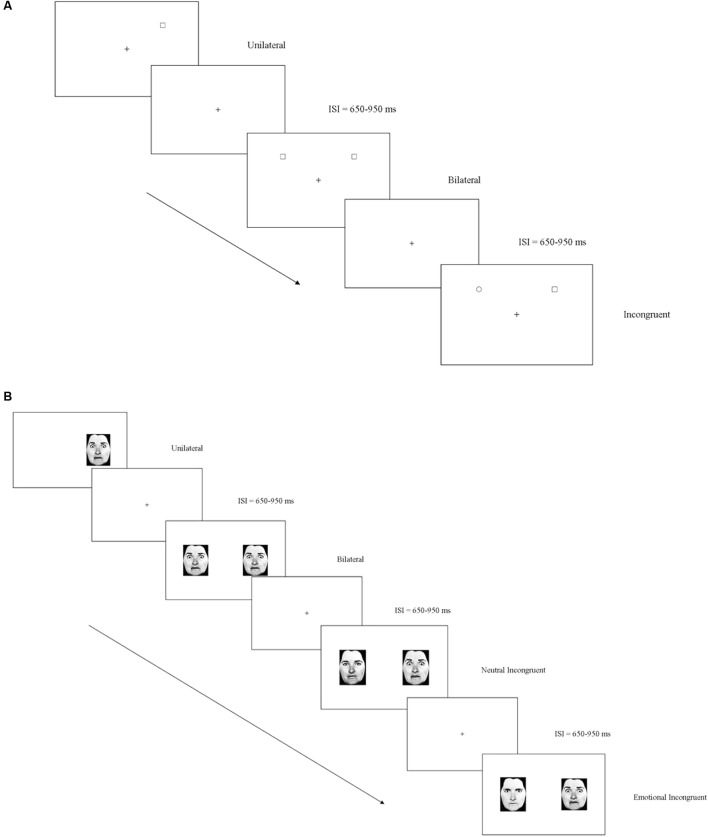
**(A)** Non-emotional task: examples of the three experimental (unilateral, bilateral, incongruent) conditions when the target was a square. **(B)** Emotional task: examples of the four experimental (unilateral, bilateral, neutral incongruent, emotional incongruent) conditions when the target was the emotion of fear.

The stimuli were presented in black against a white background. A target (an empty square/an empty triangle) was presented in the upper or lower visual field in the following conditions: (1) in the *unilateral condition*, the target was presented on the right OR left of a fixation cross; (2) in the *bilateral condition*, the target was presented simultaneously on the right AND left of the fixation cross; (3) in the *incongruent condition*, the target was presented on the right OR left of the fixation cross while a distractor (an empty circle) was presented concurrently on the opposite side of the visual display. Moreover, catch trials (representing the *no-go* condition) in which a distractor (an empty circle) was presented unilaterally, bilaterally, or together with another distractor, were implemented in the experiment. An answer in these conditions represents a false alarm, since participants should not have provided any answer. The square and the triangle (the target) were shown independently in different blocks. Participants were required to respond as soon as possible after they noticed the target. The stimuli stayed until the participants answered or for duration of 1500 ms. The inter-stimulus interval varied randomly between 650 and 950 ms (**Figure 2A**). For each condition (unilateral, bilateral, incongruent), 32 valid trials and 16 catch trials were presented in 4 blocks (ABBA: square, triangle, triangle, square). Overall, 576 trials were administered. There was a 2- to 3-min break between blocks. *Accuracy* (% hits - % false alarms) and *Reaction Time* (*RT*) from stimuli onset were recorded for valid trials.

### Emotional Task

Photographs of male and female faces (Ekman and Friesen, 1976) with either an angry, a fearful, or a neutral expression, were presented in four different conditions: (1) in the *unilateral condition*, the target (anger/fear) was presented on the right OR left of a fixation cross; (2) in the *bilateral condition*, the target was presented simultaneously on the right AND left of the fixation cross; (3) in the *neutral incongruent condition*, the emotion target was presented on the right OR left of the fixation cross along with a neutral expression; (4) in the *emotional incongruent condition*, the target was presented on the right OR left of the fixation cross along with a different emotion. Moreover, in the catch trials, a distractor (represented in half the trials by neutral stimuli and in the other half by a contrasting emotion) was presented unilaterally, bilaterally, or in opposition to a neutral and another emotion stimuli. The emotions of fear and anger were studied independently in different blocks. Participants were required to respond as soon as they noticed the target. The stimuli stayed until the participants responded or for a duration of 1500 ms. The inter-stimulus interval varied randomly between 650 and 950 ms (**Figure 2B**). For each condition (*unilateral, bilateral, neutral incongruent; emotional incongruent*), 32 valid trials and 16 catch trials were presented in 4 blocks (ABBA: anger, fear, fear, anger). Overall, 768 trials were administered. There was a 2- to 3-min break between blocks. *Accuracy* (% hits – % false alarms) was measured. The negative scores on accuracy indicated a higher number of false alarms, meaning that the subject mislabeled an emotion stimulus as the target. Moreover, *Reaction Time* (*RT*) from stimuli onset was recorded relative to valid trials.

### Analysis

The analysis was conducted by FS (author of the present manuscript). The scores of each psychological questionnaire were computed according to the seminal articles (Macor et al., 1990; Derogatis and Savitz, 2000; Ghisi et al., 2006; Todarello and Pace, 2010). For the non-emotional task, the data were collapsed together for the upper and the lower visual fields as well as for those relative to the square and the triangle. Regarding the control group’s RT, 1.2% of valid trials were eliminated due to omissions; in other words, when individuals did not erroneously provided any answer and then no information about accuracy or RT was available for the successive analyses. In terms of the emotional task, the emotions of anger and fear were studied independently. Concerning the control group’s RT, 12.62% of valid trials for anger and 7.7% of valid trials for fear were eliminated due to omissions. The two patients’ scores for each psychological subscale as well as for the experimental data were compared to the means and the SDs of the control group using Crawford’s *t*-test for single cases (Crawford and Howell, 1998; Crawford et al., 2010).

## Results

### Case #1

#### Psychological Assessment

Patient 1 reported a significantly higher number of symptoms in the sub-scale relative to *somatization* (SCL-90) [*t* = 4.668; *p* < 0.001; 95% CI = 4.760 (3.36–6.14)] compared to the control group (**Table 2**). No other difference emerged [*p* > 0.05].

**Table 2:** For each psychological subscale of the administered psychological questionnaires, case #1’s and case #2’s scores are reported and contrasted with the control group’s means and standard deviations.

Psychological questionnaires	Max score	Control group M (*SD*)	Case #1 score	Case #2 score
Beck Depression Inventory	63	6.04 (4.8)	1	0
**State-Trait Anxiety Inventory**				
State	80	34.32 (8)	32	23
Trait	80	36.36 (9.37)	27	25
**Symptom Checklist 90-R**				
Somatization	4	0.31 (0.25)	1.5^∗∗^	2.83^∗∗^
Obsessive-compulsive	4	0.61 (0.56)	0.5	0.8
Interpersonal sensitivity	4	0.51 (0.58)	1.2	0
Depression	4	0.53 (0.51)	0.23	0.31
Anxiety	4	0.39 (0.34)	0.6	0.60
Hostility	4	0.44 (0.55)	0.3	0.17
Phobic Anxiety	4	0.11 (0.21)	0	0
Psychotic Paranoid Ideation	4	0.33 (0.53)	0.6	0.1
Paranoid Ideation	4	0.72 (0.74)	1.5	0.17
**Toronto Alexithymia Scale 20**				
Difficulty identifying feelings	28	11.52 (4.83)	7	15
Difficulty describing feelings	16	9.12 (3.46)	14	4
Externally oriented thinking	16	9.52 (3.37)	15	8


*^∗^*p* < 0.05; ^∗∗^*p* < 0.001; M, mean; SD, standard deviation. For more details, refer to the section of the “Results.”*

#### Non-emotional Task

**FIGURE 3:**
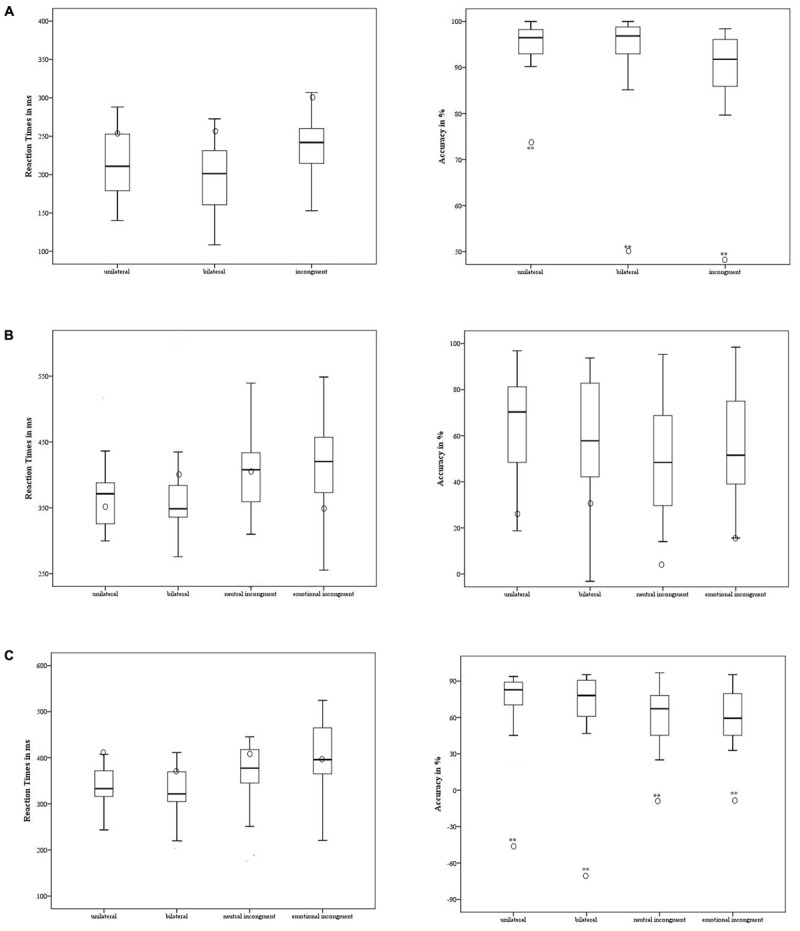
Case #1’s behavioral performance in the non-emotional task **(A)**, emotional task – anger **(B)**, and emotional task – fear **(C)** was contrasted with the control group. The means of accuracy (right side) and RT (left side) are reported for case #1 (in circle) and the control group (the minimum, the lower quartile, the median, the upper quartile, and the maximum are shown). ^∗^*p* < 0.05; ^∗∗^*p* < 0.001.

Patient 1 showed a significantly lower level *of Accuracy* in the unilateral- [*t* = 4.94; *p* < 0.001; 95% CI = -4.175 (-5.4 to -2.93)], bilateral- [*t* = 9.1; *p* < 0.001; 95% CI = -9.29 (-11.92 to -6.64)], and incongruent conditions [*t* = 5.67; *p* < 0.001; 95% CI = 5.78 (-7.45 to -4.1)] compared to the control group. The participant did not differ significantly from the control group in *RT* [*p* ≥ 0.2] (**Figure 3A**).

#### Emotional Task

Regarding the experimental conditions in which the target was the emotion of anger, the participant showed a similar level of *Accuracy* [*p* ≥ 0.8] and *RT* [*p* ≥ 0.29] to the control group (**Figure 3B**).

The participant had a significantly lower level of *Accuracy* in the emotion of fear compared to the control group in all the experimental conditions [unilateral *t* = 7.53; *p* < 0.001; 95% CI = -7.72 (-9.92 to -5.51); bilateral *t* = 6.7; *p* < 0.001 95% CI = -6.9 (-8.81 to -4.92); neutral incongruent *t* = 3.59; *p* < 0.001; 95% CI = -3.66 (-4.76 to -2.55); and emotional incongruent *t* = 3.63; *p* < 0.001; 95% CI = -3.7 (-4.81 to -2.58)] (**Figure 3C**). Notably, the accuracy was negative across all the experimental conditions: the participant showed a higher number of false alarms and labeled the stimuli erroneously as fearful, specifically when they were presented unilaterally or bilaterally. When the stimulus was contrasted with a neutral expression or with another emotion, the errors decreased. No difference emerged with respect to the control group’s performance [*p* ≥ 0.09] in *RT.*

### Case #2

#### Psychological Assessment

Patient 2 reported a significantly higher number of symptoms on the *somatization* scale (SCL-90) [*t* = 9.884; *p* < 0.001, 95% CI = 10.08 (7.21–12.93)] compared to the control group. No other difference emerged [*p* > 0.05] (refer to **Table 2**).

#### Non-emotional Task

**FIGURE 4:**
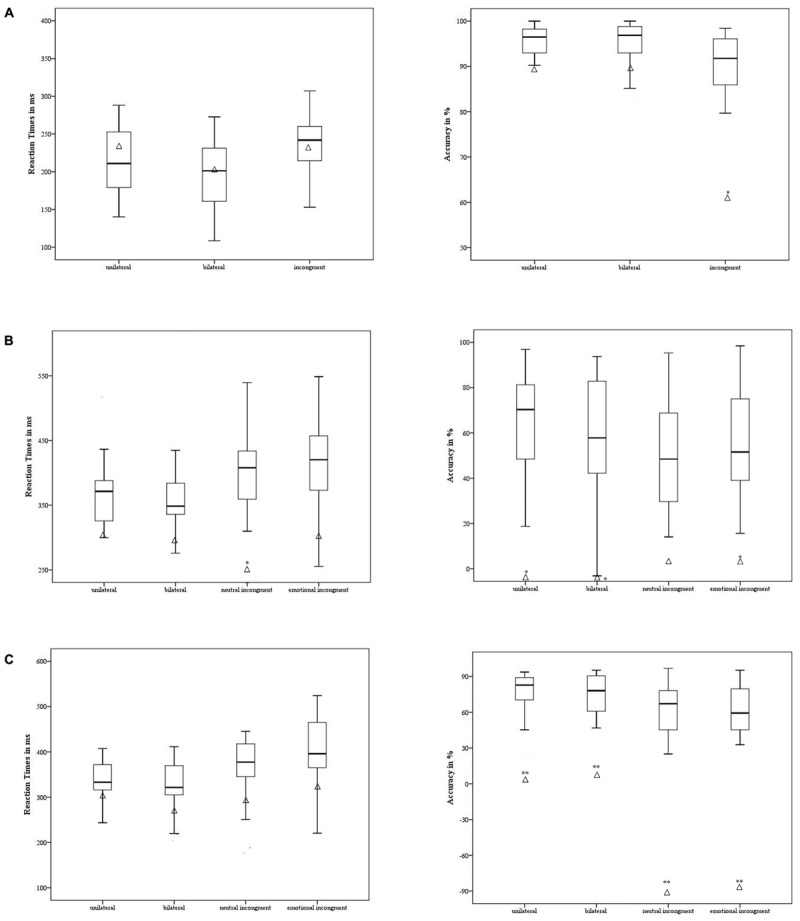
Case #2’s behavioral performance in the non-emotional task **(A)**, emotional task – anger **(B)**, and emotional task – fear **(C)** was contrasted with the control group. The means of accuracy (right side) and RT (left side) are reported for case #2 (in triangle) and the control group (the minimum, the lower quartile, the median, the upper quartile, and the maximum are shown). ^∗^*p* < 0.05; ^∗∗^*p* < 0.001.

Patient 2 had a significantly lower level *of Accuracy* only in the incongruent condition [*t* = 3.03; *p* < 0.002, 95% CI = 3.36 (-4.38 to -2.33)] when contrasted with the control group, as shown in **Figure 4**. Moreover, no difference emerged in *RT* between the participant’s and the control group’s performance [*p* ≥ 0.47] (**Figure 4A**).

#### Emotional Task

Regarding the experimental conditions in which the target was the emotion of anger, the participant showed a lower level of *Accuracy* in the unilateral [*t* = 2.48; *p* = 0.02 95%, CI = -2.53 (-3.34 to -1.71)], bilateral [*t* = 2.13; *p* = 0.04, 95% CI = -2.18 (-2.92 to -1.44), and emotionally incongruent [*t* = 2.07; *p* = 0.04, 95% CI = -2.11 (-2.81 to -1.39)] conditions but not in the neutral incongruent [*p* = 0.08] condition. Specifically, when the anger stimuli were showed unilaterally and bilaterally, the participant mislabeled the emotion, as suggested by the negative scores. The patient was faster in *RT* compared to the control group only in the neutral incongruent condition [*t* = 2.47; *p* = 0.02; 95% CI = -2.47 (-3.26 to -1.67] (**Figure 4B**).

Regarding the emotion of fear, the participant showed a lower level of *Accuracy* compared to the control group in all experimental conditions [unilateral *t* = 4.01; *p* < 0.001; 95% CI = -4.09 (-5.3 to -2.87); bilateral *t* = 4.1; *p* < 0.001; 95% CI = -4.18 (-5.42 to -2.94); neutral incongruent *t* = 7.5; *p* < 0.001; 95% CI = -7.65 (-9.83 to -5.46); and emotionally incongruent *t* = 7.33; *p* < 0.001; 95% CI = -7.48 (-9.61 to -5.34)]. Again, the accuracy was negative across the experimental conditions. No difference emerged in terms of the control group’s performance [*p* ≥ 0.26] in *RT* (**Figure 4C**).

## Discussion

In this case-controls study, we investigated the ability of two individuals with NOVL to recognize the emotions of fear and anger expressed by others. Our results clearly showed that both individuals showed a certain difficulty in efficiently recognizing the emotion of fear. They also mislabeled other emotional stimuli, judging them as fearful. On the other hand, they described themselves as completely proficient in their emotional capability: no sign of alexithymia was reported in the TAS – 20 (Todarello and Pace, 2010).

The first result we discuss is the difficulties of the two participants to efficiently recognize the emotion of fear. Why would fear be erroneously processed in NOVL? To answer this question, we might take in account two facts. Firstly, the conversion symptom is described as a defensive reaction to upsetting, traumatic, and potentially frightening situations. Secondly, fear is an emotion evoked rapidly by situations that are subjectively perceived (even though not consciously) as dangerous (Adolphs, 2008). However, just as the detection of fearful stimuli from the environment is crucial for surviving, so is the extinction of this emotion when the threating stimuli disappear (Barad et al., 2006). Indeed, considering the environment as an excessive threat would cause a constant neurophysiological fear-related activation with very high levels of stress (van der Kolk, 1997). One survival mechanism to overcome this impasse might be to turn off the sensitivity to read the emotion of fear in others’ faces as the main vehicle of alert (Adolphs, 2008) or to read one’s own arousing, bodily panic-related sensations as symptoms of mental illness (Demartini et al., 2014), because of alexithymia. Thus, in NOVL, individuals might have a specific difficulty in recognizing the emotion of fear as a *defensive mechanism*. This mechanism was already hypothesized for other forms of psychopathology (Maren et al., 2013). Among these disorders, the most representative is the post-traumatic stress disorder: affected individuals generally show a reduced accuracy or a decreased sensitivity to fearful expressions (Rougemont-Bucking et al., 2011; Poljac et al., 2011) in emotion recognition tasks. This impairment might be due to a dysfunction in that cerebral network (hippocampus, amygdala and medial prefrontal cortex) involved in the generation of a context-dependent behavior based on previous experiences (van der Kolk, 1997; Maren et al., 2013). Indeed, a reduced medial prefrontal cortex activity (Bremner et al., 1999) associated with an exaggerated amygdala response to general negative stimuli in post-traumatic stress disorder (Bremner et al., 1999; Rauch et al., 2000; Bryant et al., 2008) was observed in neuroimaging studies; moreover, a similar cerebral mechanism in terms of greater arousal activity (Seignourel et al., 2007; Bakvis et al., 2009a,b) as well as abnormalities in the amygdala and its interactions with other cerebral areas (Voon et al., 2010; Aybek et al., 2015) has also been reported in conversion disorder, motor variant. Considering that both individuals affected by NOVL showed a certain difficulty in recognizing efficiently the emotion of fear, a similar mechanism for this psychopathological condition might be hypothesized, requiring further investigation in which the behavioral results are linked to the cerebral functional activity. We might suggest focusing on the role of the amygdala, instead of occipital (visual) areas, as previously done by Werring et al. (2004) and Schoenfeld et al. (2011).

According to the behavioral results, the two individuals affected by NOVL not only showed a selective difficulty in recognizing efficiently the emotion of fear, but they also mislabeled other emotional stimuli, judging them as fearful. This behavior might be explained according to two different lines of results. The first involves the phenomenon of *negative bias* toward emotional stimuli, generally reported in several psychopathological conditions, such as depression (Mandal and Bhattacharya, 1985; Gur et al., 1992), eating disorders (Harrison et al., 2010), as well as schizophrenia (Kohler et al., 2003) and borderline personality disorder (Dyck et al., 2009): individuals erroneously misidentify neutral or positive stimuli as negatively valenced; this misattribution may in part underline global difficulties and inappropriate behavior in social interactions (Kohler et al., 2003). The performance of the two individuals with NOVL in the emotional task might be the expression of this negative bias.

Secondly, the mislabeling of emotion stimuli as fearful might also be related to the neuroanatomical fear-related network (the direct subcortical inputs from the thalamus to the amygdala, bypassing the slower conscious analysis in the ventral visual) (Vuilleumier et al., 2003). This network quickly primes the perception of fear-related stimuli to allow for a fast response (Butler et al., 2009; Norton et al., 2009; West et al., 2010). This faster processing of the fearful stimulus is reflected in the experimental behavior: responses to negative stimuli tend to be quicker than responses to positive stimuli (Ohman et al., 2001; Leppanen et al., 2003; Hugenberg, 2005) when participants must simply perceive stimuli without making any cognitive judgments about them. However, if the fear-related processing is impaired, it may cause an early mislabel of a neutral expression as fearful, in other words before it is can be completely processed in the primary visual areas. This mechanism, which has been preliminarily suggested in schizophrenia (Premkumar et al., 2008; Habel et al., 2010; Bedwell et al., 2013), might explain the performance of the two individuals with NOVL in the emotional task. Future research using neuroimaging and neurophysiological techniques might support this hypothesis.

Despite the behavioral results, both individuals affected by NOVL judged themselves as proficient in the emotional processing: in fact, no sign of alexithymia was reported in the Toronto Alexithymia Scale 20 (TAS-20) (Bagby et al., 1994; Todarello and Pace, 2010). Moreover, the two participants did not report abnormal levels of anxiety (of which fear represents the core) in the State-Trait Anxiety Inventory (STAI) (Spielberger et al., 1983; Macor et al., 1990). Thus, a discrepancy emerged between the behavioral results, showing an aberrant facial emotion recognition of fear, and the conscious self-judgment of emotion recognition capability. Even though the relation between these two components remains to be explored, as well as the emotional processing of other primary emotions, we might suggest a failure of self-reported scales to discern alexithymia when applied in this clinical population. Indeed, the self-reports measure emotional processing and internal feelings by asking individuals to quantify and describe their emotions and feelings explicitly. However, the assumption is that they might show an inability to recognize and verbally describe their emotions, an impaired capacity for empathy and self-insight, or an inability to discriminate between emotional states and bodily sensations (Frawley and Smith, 2001). Rather, when individuals are expected to be alexithymic, indirect measures of emotional processing might be more suitable to avoid false negative cases.

In the non-emotional task, in which participants were asked to recognize geometrical shapes, different results between the two patients were observed. The case #1 showed lower level of accuracy in all experimental conditions respect to the control group; instead the case #2 only in the incongruent one, in which an unattended stimulus competed with the target (de Gelder et al., 2001). Heterogeneous, but limited results are reported in literature about dysfunctional primary sensory process in NOVL: Schoenfeld et al. (2011) and Becker et al. (2013) reported unaltered visual cortex responses in presence of a visual target; however, altered amplitude of the visual evoked potential N1, which reflects the operation of the discrimination process within the focus of attention (Vogel and Luck, 2000), was observed (Schoenfeld et al., 2011). Thus, our results cannot be a supporting or a contrasting evidence about preserved perceptual ability in NOVL, specifically if we take in account the reduced number of participants assessed and the absence of any neurophysiological or neuroimaging measure of cerebral activity during the task. Of course, future investigation is necessary to clarify the functionality of visual primary areas in NOVL, with the aim to recognize possible broader neural and cognitive difficulty (and then not completely limited to the emotional processing), as yet suggested for conversion disorder, motor variant in which alteration in the activity of the primary motor and sensory cortex, in addition to that of the limbic circuit, was observed (Boeckle et al., 2016 for a review).

## Conclusion

This preliminary investigation reports, for the first time in the literature, an interesting mismatch between the explicit (i.e., the subjective evaluation of their own emotional capability) and the implicit (i.e., the behavior in experimental paradigm) components of the emotional processing in participants with NOVL. Of course, future investigation with an enlarge numbers of participants and with more strictly inclusive and exclusive criteria, specifically in relation to the clinical history and the psychiatrist evaluation, is necessary. However, this report might represent a starting point for more detailed research to investigate how visual problems, when not directly related to physical damage, may (or may not) be related to psychological factors as well as alexithymia, and might be conceptualized as a maladaptive reaction construct in unelaborated traumatic experiences (Franzoni et al., 2013), as already reported in functional motor symptoms (Demartini et al., 2014).

## Author Contributions

FS: conceived the study, conducted the experimental part and the statistical analyses, and wrote the paper. LM: enrolled and evaluated the patients with NOVL and reported their description in the manuscript. GC: conceived and conducted the psychological evaluation and revised the manuscript. AM: supervised the experimental part and the neurological assessment of the patients. SBM: supervised the evaluation of the patients with NOVL and revised the manuscript. EM: conceived the study, supervised the study, and revised the manuscript.

## Conflict of Interest Statement

The authors declare that the research was conducted in the absence of any commercial or financial relationships that could be construed as a potential conflict of interest.
